# The unacknowledged co-author: LLM-mediated reasoning in plant metabolomics and its systematic blind spots

**DOI:** 10.3389/fpls.2026.1832678

**Published:** 2026-05-19

**Authors:** Sara Postacchini, Patricia Pacheco-Ruiz, José G. Vallarino, Luca Mazzoni

**Affiliations:** 1 Department of Agricultural, Food and Environmental Sciences (D3A), Università Politecnica delle Marche, Ancona, Italy; 2 Instituto de Hortofruticultura Subtropical y Mediterránea “La Mayora”, Departamento de Biología Molecular y Bioquímica, Universidad de Málaga-Consejo Superior de Investigaciones Científicas (IHSM-UMA-CSIC), Málaga, Spain

**Keywords:** biological interpretation, hallucination, large language models, metabolite annotation, reproducibility, untargeted metabolomics, validation

## Introduction

A growing fraction of plant metabolomics research appears to be shaped by a collaborator that never appears in the author list. Large language models (LLMs) are now routinely consulted by researchers to interpret untargeted metabolomic datasets, suggesting candidate identities for unresolved features, proposing pathway narratives that connect disparate signals, generating hypotheses that link statistical patterns to biological mechanisms. Based on informal accounts and community discussion, this practice appears to be increasingly common and, in most cases, undisclosed; no validation standard has yet been established.

The situation differs fundamentally from the use of LLMs as writing assistants, a practice that journals have begun to regulate. When a researcher asks an LLM to suggest what a feature at m/z 611.16 in a strawberry extract might be, and the model confidently returns “rutin (quercetin-3-O-rutinoside), a flavonol glycoside commonly found in Rosaceae”, that answer enters the researcher’s reasoning chain. Any researcher who has submitted an unresolved feature list to ChatGPT, Gemini or other LLM will recognize this pattern: the response is immediate, structurally specific, and delivered without qualification. If it aligns with prior expectations, it may be accepted without orthogonal verification. If it reaches the manuscript as a tentative annotation, a pathway discussion, or a mechanistic hypothesis, it becomes part of the scientific record without any transparent audit trail.

This is not a hypothetical concern. Lu et al. developed MetaBench, a systematic benchmark testing factual metabolomics knowledge across 25 LLMs using approximately 8,000 structured queries drawn from HMDB, KEGG, PathBank and MetaboLights (Lu et al., 2025). MetaBench answers a necessary question: how accurately do LLMs retrieve established metabolomic facts? Its findings are instructive but also delimit its scope. LLM accuracy declines sharply for poorly annotated metabolites, with performance gaps exceeding 20 percentage points between well-studied and sparsely characterized compounds, and cross-database identifier grounding remains a critical failure point even with retrieval augmentation. Yet MetaBench operates in a closed-answer regime: it asks, for instance, whether a model correctly identifies the molecular weight of quercetin as 302.24 Da. Our concern is different. We address what happens when a researcher submits ten unresolved features from a strawberry LC-MS dataset and accepts the model’s suggestion that feature 47 is quercetin without orthogonal verification. MetaBench does not capture this informal, unstructured co-reasoning applied to high-dimensional, largely unannotated experimental data. The two problems are complementary; conflating them would obscure both.

## Anatomy of the biases

None of the three failure modes described below is unique to large language models. Canonical overrepresentation has a human analogue in confirmation bias and the familiar reliance on well-characterized compounds; confident hallucination mirrors the documented human tendency toward overconfident identification in the absence of sufficient evidence; narrative fabrication resembles the construction of *post hoc* mechanistic stories familiar to any researcher who has drafted a discussion section. The concern is not that these biases exist, but that LLMs amplify them through scale, obscure them through the absence of a deliberative trace, and naturalize them behind a surface of computational objectivity. A human researcher can be asked how they reached a conclusion; an LLM cannot.

Three systematic failure modes emerge when LLMs are applied to the interpretation of untargeted plant metabolomics data. The first is canonical overrepresentation. LLMs are trained predominantly on published literature, which is itself biased toward well-characterized metabolites. When asked to interpret a feature list from a plant extract, models disproportionately suggest compounds that appear frequently in databases and publications: chlorogenic acid, rutin, catechin, and kaempferol glycosides. The MetaBench results confirm this structurally: model accuracy is substantially higher for metabolites with low HMDB identifiers (older, extensively annotated compounds) and degrades monotonically for those with high identifiers (recent, sparsely annotated entries) (Lu et al., 2025). Rarer or poorly characterized secondary metabolites, which may constitute the majority of biologically relevant variation in a given experiment, are systematically underrepresented in LLM outputs. The result is an interpretive gravitational pull toward the known, which reinforces existing annotation biases rather than challenging them. Recent reviews of LLM applications in plant breeding, where metabolomic data is positioned as one component of multi-omics integration (Yoosefzadeh-Najafabadi, 2025), illustrate the broader pattern: generic limitations such as training data bias and interpretability are acknowledged, but the failure modes specific to metabolomic annotation—hallucination in chemical space, systematic preference for canonical compounds, narrative coherence prioritized over mechanistic fidelity—are not engaged with.

The second failure mode is confident hallucination in poorly characterized chemical space. LLMs do not signal uncertainty proportionally to the difficulty of the query. A request to identify a well-studied primary metabolite and a request to identify an obscure acylated flavonoid glycoside receive answers delivered with equivalent confidence. This scenario is familiar to anyone working with untargeted data in *Fragaria*: consider a feature at m/z 757.22 detected in strawberry fruit extract, for which a plausible candidate would be an acylated pelargonidin diglycoside, a compound class reported in *Fragaria* but largely absent from spectral databases. An LLM, lacking spectral evidence, may nonetheless assign a structurally specific identity based solely on mass and taxonomic context, generating a false anchor that distorts downstream pathway analysis. Wang et al. have documented this pattern in bioinformatics broadly: LLMs generate plausible but fabricated associations between biological entities, and the fabrication rate increases in knowledge-sparse domains (Wang et al., 2024). Plant secondary metabolism, with its combinatorial diversity of glycosylation, acylation and methylation patterns, is precisely such a domain.

The third failure mode is narrative fabrication. The generation of coherent but unsupported mechanistic stories connecting unannotated features to known biological pathways. This is arguably the most dangerous bias because it is the most useful to the researcher. A list of differentially accumulated features is difficult to interpret; a narrative explaining that “the upregulation of features in the phenylpropanoid molecular weight range, combined with the downregulation of features tentatively assigned to the flavonol branch, suggests a metabolic diversion toward hydroxycinnamic acid derivatives under stress conditions” is immediately compelling. Anyone who has used an LLM to contextualize a set of differentially accumulated features will recognize the fluency of these outputs and the temptation to adopt them as working hypotheses. Yet such narratives may be constructed from tentative annotations, assumed pathway topologies, and pattern-matching against training data rather than from any experimental evidence specific to the dataset at hand.

## The reproducibility problem

The biases described above would be concerning even if they were transparent. What makes them a threat to the integrity of the metabolomics literature is their invisibility.

When a researcher uses a statistical tool, the tool, its version, and its parameters are reported in the methods section. When a researcher uses an LLM to guide biological interpretation, this step is, to our knowledge, rarely if ever reported. There is no methods section entry for “pathway hypotheses were developed in dialogue with GPT-4.” There is no supplementary file logging the prompts, the model version, or the specific outputs that were accepted versus rejected.

This opacity creates a reproducibility problem of a new kind. Two researchers analyzing the same dataset can, in principle, reach different biological conclusions, not because their analytical pipelines differ, but because their LLM interactions differed: different prompts, different models, different stochastic outputs. Such divergence would be real but untraceable. It is compounded by the fact that LLM outputs are not static: a query submitted to the same model months apart may return different answers because the model has been updated or replaced, rendering the biological interpretation of a dataset dependent on a tool that is non-deterministic, non-versioned, and non-reproducible even in principle.

Liu et al. have begun to explore LLM-assisted metabolite annotation as a formalized workflow (Liu et al., 2025), and PlantGPT represents an attempt to build domain-specific models for plant biology (Zhang et al., 2025). These efforts are valuable precisely because they make the LLM’s role explicit and auditable. The danger lies not in the use of LLMs *per se*, but in their use as invisible reasoning partners whose contributions escape peer review.

## A framework for disclosure and validation

**Figure 1:**
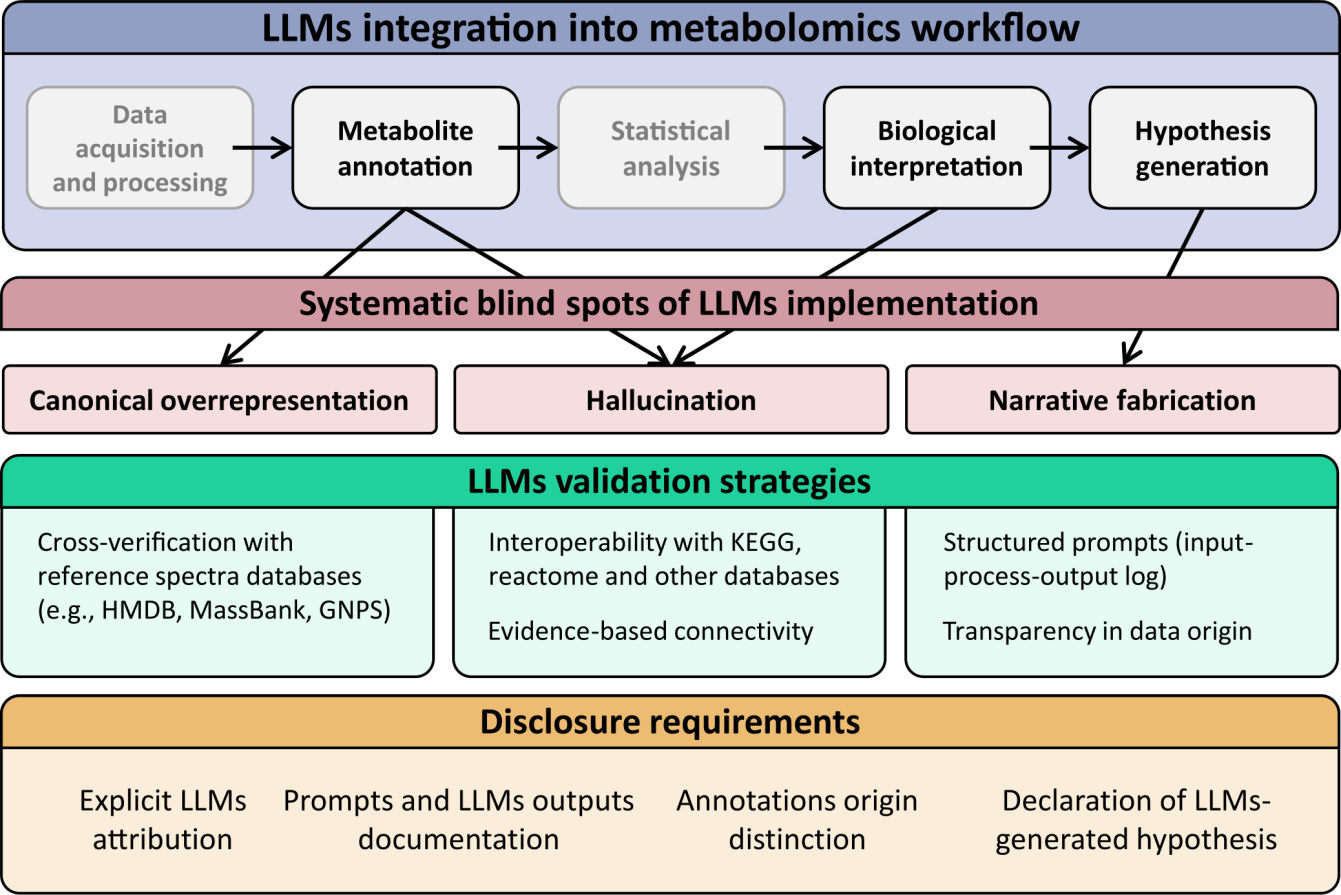
Integration of large language models (LLMs) into the plant metabolomics workflow. The top band shows a typical interpretation pipeline; steps at which LLMs intervene are highlighted (metabolite annotation, biological interpretation, hypothesis generation), while steps where they do not meaningfully operate are shown in gray. Three systematic failure modes arise from these intervention points (middle band): canonical overrepresentation and confident hallucination at metabolite annotation, further hallucination risk at biological interpretation, and narrative fabrication at hypothesis generation. The lower bands present the proposed mitigation framework: validation strategies, including cross-verification against spectral databases (e.g., HMDB, MassBank, GNPS), interoperability with curated pathway resources (e.g., KEGG, Reactome), and structured prompt logging; and disclosure requirements, covering explicit LLM attribution, documentation of prompts and outputs, distinction between database-derived and LLM-suggested annotations, and flagging of LLM-generated hypotheses.

We do not argue that LLMs should be excluded from metabolomics research. They are too useful, and the prohibition would be unenforceable. We argue instead that their use in biological interpretation must be governed by minimum standards of disclosure and validation (**Figure 1**).

Disclosure requires four elements: an explicit statement in the methods section that LLM-assisted reasoning was used for biological interpretation, specifying the model, version and access date; deposition of the prompts and raw LLM outputs as Supplementary Material; a clear distinction in the text between annotations derived from spectral matching against reference databases and annotations suggested or reinforced by LLM interaction; and identification of which biological hypotheses or pathway narratives were generated or substantially shaped by LLM outputs.

Validation requires, at minimum, two orthogonal checks. Any metabolite identity suggested by an LLM and used in the manuscript must be verified against at least one spectral database (HMDB, MassBank, GNPS) with reported match scores. Any pathway narrative generated with LLM assistance must be cross-referenced against at least one curated pathway database (KEGG, MetaCyc, PlantCyc) with explicit notation of which steps are supported by database evidence and which are inferred.

These requirements are modest. They do not demand that researchers avoid LLMs, nor that they perform additional experiments. They demand only that the role of the LLM in the reasoning process be visible to reviewers and readers, and that the most basic forms of orthogonal verification be documented. Allwood et al. catalogued the technical challenges facing plant metabolomics before LLMs entered the interpretive workflow (Allwood et al., 2021); the addition of an opaque reasoning tool to an already under-standardized field makes transparency not optional but urgent.

The alternative is to accept that a growing fraction of the plant metabolomics literature rests on biological claims whose provenance cannot be evaluated. That is not a standard any field should find acceptable.
